# Letter in response to “The gender affirming model of care is competent, ethical medical practice”

**DOI:** 10.1177/10398562251413166

**Published:** 2026-01-02

**Authors:** Andrew James Amos

**Affiliations:** Division of Tropical Health and Medicine, College of Medicine and Dentistry, James Cook University, Townsville, QLD, Australia

Dear Editor,

I thank Dr Agapoff for his comment disagreeing with my article asserting that gender affirming care (GAC) is incompatible with competent, ethical medical practice. While applauding the courage of a psychiatrist with trans experience to take a public position on GAC, it is necessary to acknowledge the possibility that his judgement might be influenced by personal interest.^1^ As noted below, it has been argued that biases caused by personal trans experience played a determinative role in the substitution of gender dysphoria for gender identity disorder in the DSM-5 itself.^2^

This is most evident in the failure to address either of my two principal arguments. I propose GAC is incompetent and unethical because it relies upon medical diagnoses based on subjective self-report without any acceptable empirical evidence of reliability or validity, created to achieve political goals and not to address psychiatric realities^2^; and that it actively prevents understanding of the pathological processes underlying dissociation between actual and perceived sex which is the only necessary and sufficient feature of trans identification.^3^

Advocates for GAC, including Dr Agapoff, provide no account of the phenomenology of trans experiences, no description of how healthy development results in dissociation between actual and perceived sex, and no means of differentiating healthy from pathological causes of gender diversity. In my opinion this is because the phenomenology of trans identity is indistinguishable from that of delusions caused by psychosis. Following Jaspers, trans identification is an ununderstandable experience that is phenomenologically irreducible (Table 1).^4^ Any competent outline of a method to differentiate trans identification from psychosis on the basis of phenomenology would confirm they can’t be differentiated.

**Table 1.** Applying Jaspers’ phenomenological account of psychosis to trans experience (italics in original)

	*Delusion-like ideas*	*Delusions proper*
	“We can then distinguish two large groups of delusion according to their *origin*:”	
Jaspers’ description of the two forms of delusions^4^ (p. 96)	“…one group *emerges understandably* from preceding affects, from shattering, mortifying, guilt-provoking or other such experiences, from false-perception or from the experience of derealisation in states of altered consciousness etc.”	“The other group is for us *psychologically irreducible*; phenomenologically it is something final. …should the patient…retain his false judgement of reality in spite of reflection and with absolute certainty – overcoming indeed any initial doubts he may have had – then we are dealing with delusion proper: such a belief is no longer understandable in terms of hallucination alone.”
Examples of trans experience correlating with the two forms of delusion	Identification with the opposite sex in the context of homosexuality in a society where transsexuality is more acceptable than homosexuality, such as modern-day Iran.	Identification with the opposite sex as the result of “being born in the wrong body”. It should be noted that this demotic statement of the aetiology of transgender identity is necessary because there is no clinical description of the aetiology in any of the major transgender guidelines or standards of care.

Lawrence usefully contrasts the known physical pathologies that cause the gender confusion of disorders of sex development with the unknown and unexamined psychopathologies that dissociate actual from perceived sex in trans identifying people with stereotypical binary genetic sex. She uses the distinction to illustrate the motivations that compelled the activist clinicians in the DSM-5 sub-workgroup on gender identities to create gender dysphoria de novo to avoid the narcissistic injury to late onset MtF transsexual patients associated with “being reminded that they have chosen to live in a gender role that is inconsistent with their biological sex”^3^ (p. 1264).

Competent, ethical medical management of trans patients is impossible when GAC rules out a priori that mental illness plays a role in the development of dissociations between actual and perceived sex. It is characteristic that Agapoff cites Wanta et al. as evidence that there is no relationship between trans identity and psychosis rather than reflect on the fact that the ∼5% rate of schizophrenia/schizoaffective disorder in their sample was around ten times the expected prevalence, and the 11% rate of bipolar disorder was two to three times that expected.^5^

The lack of empirically demonstrated psychological treatments for problems associated with trans identities is largely due to the small number of patients and the extraclinical preferences of the doctors who have treated them. Just as it was thought that borderline patients were untreatable before DBT,^6^ the main barrier to integration of dissociated sex is the suppression of research into novel forms of psychotherapy suitable for this specific population.

We have no valid and reliable clinical labels for the psychopathologies associated with dissociation between actual and perceived sex, but their high morbidity and mortality is unquestioned. Preventing the search for an understanding of the mechanisms and exploring potential cures for these types of dissociation is analogous to preventing the work done by Marsha Linehan to help borderline patients integrate their fragmented selves.

Space preventing a full accounting of the issues here, I repeat the offer to debate Dr Agapoff, or any GAC advocate, in the live or online forum of their choosing.
